# 

**Published:** 1932

**Authors:** 


### CONTENTS.

| | Page |
|---|---|
| **Mental Deficiency: An Analysis of the Mental, Physical and Medical Characteristics of a Group of One Hundred and Sixty-Two Adult Feeble-Minded Women.** By Richard J. A. Berry, M.D., F.R.C.S., F.R.S.E. | 177 |
| **Medical Education.** By C. E. S. Flemming, M.R.C.S., L.R.C.P. | 199 |
| **Dysphagia with Anæmia, with Reports on Five Cases.** By E. Watson-Williams, M.C., Ch.M., F.R.C.S. | 209 |
| **Albuminuria in Pregnancy.** By R. S. S. Statham, M.D., Ch.M. | 219 |
| **Albuminuria in Pregnancy.** By J. A. Nixon, C.M.G., M.D., F.R.C.P. | 229 |
| **Notes on Female Circumcision as Practised by the Ameru.** By H. W. Brassington, M.B., Ch.B. | 237 |
| **The British Pharmacopœia, 1932.** By A. L. Taylor, M.P.S. | 241 |
| **Reviews of Books** | 246 |
| **Editorial Note** | 250 |
| **Obituaries:** Henry Lawrence Ormerod, M.D., B.Ch. R.U.I. | 251 |
| Frederick Graham Lilley, L.D.S., R.C.S. | 252 |
| **The Medical Library of the University of Bristol** | 253 |
| **Local Medical Notes** | 254 |

